# Epitope Mapping of Conformational V2-specific Anti-HIV Human Monoclonal Antibodies Reveals an Immunodominant Site in V2

**DOI:** 10.1371/journal.pone.0070859

**Published:** 2013-07-29

**Authors:** Luzia M. Mayr, Sandra Cohen, Brett Spurrier, Xiang-Peng Kong, Susan Zolla-Pazner

**Affiliations:** 1 Department of Pathology, New York University School of Medicine, New York, New York, United States of America; 2 Department of Biochemistry and Molecular Pharmacology, New York University School of Medicine, New York, New York, United States of America; 3 Veterans Affairs New York Harbor Healthcare System, New York, New York, United States of America; Chinese Academy of Medical Sciences, China

## Abstract

In the case-control study of the RV144 vaccine trial, the levels of antibodies to the V1V2 region of the gp120 envelope glycoprotein were found to correlate inversely with risk of HIV infection. This recent demonstration of the potential role of V1V2 as a vaccine target has catapulted this region into the focus of HIV-1 research. We previously described seven human monoclonal antibodies (mAbs) derived from HIV-infected individuals that are directed against conformational epitopes in the V1V2 domain. In this study, using lysates of SF162 pseudoviruses carrying V1V2 mutations, we mapped the epitopes of these seven mAbs. All tested mAbs demonstrated a similar binding pattern in which three mutations (F176A, Y177T, and D180L) abrogated binding of at least six of the seven mAbs to ≤15% of SF162 wildtype binding. Binding of six or all of the mAbs was reduced to ≤50% of wildtype by single substitutions at seven positions (168, 180, 181, 183, 184, 191, and 193), while one change, V181I, increased the binding of all mAbs. When mapped onto a model of V2, our results suggest that the epitope of the conformational V2 mAbs is located mostly in the disordered region of the available crystal structure of V1V2, overlapping and surrounding the α4β7 binding site on V2.

## Introduction

In 2009, the RV144 clinical vaccine trial provided the first success in the development of an effective HIV vaccine, demonstrating a 31.2% efficacy at preventing HIV-1 infection in vaccine recipients [1]. In the recently published case-control study, levels of IgG antibodies specific for the first and second variable regions (V1V2) of gp120 were shown to inversely correlate with the reduced rate of HIV-1 infection [2]. The hypothesis based on these data, that V1V2 antibodies are protective, is also supported by a recent publication reporting an increased RV144 vaccine efficacy against viruses with genetic signatures at two positions in V2 [3]. These data generated the hypothesis that the V2 region of gp120 is likely to be a promising target for vaccine development and recent HIV-1 vaccine research has increasingly focused on antibodies specific for V2. The V1V2 region can form four anti-parallel β-strands (A, B, C, and D) linked via disulfide bonds [4]; these are located at the apex of the envelope trimer [5]. Comparison of all V2 sequences in the LANL database revealed that whilst V2 loops can vary in length, the majority of amino acids are highly conserved or permit only conservative substitutions [6]. This, and the broad immunologic cross-reactivity of many human V2 mAbs with gp120s from diverse clades, suggest that the V2 region contains structurally and antigenically conserved elements [7]–[10].

V2 can bind to α4β7, an integrin that is expressed strongly on activated CD4^+^ T cells [11]. α4β7, in concert with chemokine receptors, is essential for homing of CD4^+^ T cells to the gut mucosa [12]–[14]. On activated gut CD4^+^ T cells, α4β7 has been shown to co-localize with CD4 and CCR5 in the cell membrane [14] and, whilst not required for *in vitro* viral entry or replication, α4β7 interacts with gp120 proteins from diverse HIV-1 subtypes, suggesting that this integrin may play a key role in mucosal HIV-1 transmission [14], [15]. Binding to α4β7 is mediated by a conserved tri-pepetide in the V2 loop, the LDV/I binding motif [11], which mimics the tri-peptide binding sites encoded by the natural ligands of α4β7 [16]. However, in recent studies, antibodies specific for α4β7 failed to block infection of activated CD4^+^ T cells by full-length infectious molecular clones of subtype C viruses, suggesting that differences in binding may exist between gp120 proteins and the trimeric envelopes present on virions [17].

Antibodies targeting the V2 region are found in approximately 20–45% of HIV-1 infected individuals [8], [18]–[20]. Monoclonal antibodies (mAbs) that recognize epitopes in V2 have been shown to target linear, quaternary, or conformational epitopes. The epitopes of mAbs CH58 and CH59, isolated from RV144 vaccine recipients, recognize a continuous linear region of V2, whilst the broadly neutralizing mAbs PG9 and PG16 preferentially target a highly complex, glycan-dependent quaternary epitope including parts of V1, V2 and V3 on the surface of virions and infected cells [4], [21], [22]. These two types of mAbs differ from the conformation-dependent V2 mAbs that were the initial V2 human mAbs to be characterized [7], [20].

The conformation-dependent V2 mAbs, namely mAbs 697, 830A, 1357D, 1361, 1393A, 2158, and 2297, display broad immunologic cross-reactivity with subtype A, AG, B, and C recombinant gp120 proteins and weak neutralization of Tier 1 pseudoviruses [7], [20], [23]. Initial mapping studies of mAb 697 showed that it targets highly conserved amino acids located between positions 164 and 194 in HxB2 [7], an area that includes the α4β7 tri-peptide binding motif. Competition assays suggested that the epitopes of all the conformational V2 mAbs, with the exception of mAb 2297, are similar to or overlap with that of mAb 697 [20]. This category of conformational V2 Abs is particularly interesting in that the mAbs of this type react strongly with V1V2-fusion proteins, and specifically with the MuLV gp70 fusion protein carrying the V1V2 region of subtype B strain A2. Binding of vaccinees' plasma Abs to this V1V2-fusion protein was inversely related to the risk of infection, providing the first correlate of risk in a vaccine study [2]. In contrast to the reactivity of conformational V2 mAbs with this protein, the quaternary V1V2V3-directed mAbs do not react with V1V2_Case A2_-gp70, and the RV144-derived V2 mAbs react with this reagent much more weakly or not at all [22]. Consequently, it is crucial to precisely delineate the nature of the epitope(s) recognized by the conformational V2 mAbs.

## Materials and Methods

### Ethics Statement

This study was approved by the New York University School of Medicine Institutional Review Board.

### Site-directed mutagenesis

Point mutations were introduced in the V1V2 region of plasmid pCAGGS SF162 gp160 (NIH AIDS Reagent Program) [24]–[26] using the QuikChange II XL Site-Directed Mutagenesis Kit (Stratagene, La Jolla, CA, USA), according to the manufacturer's instructions. All mutant constructs were sequenced to confirm the correct amino acid change. Plasmid pCAGGS SF162 gp160 K160N was kindly supplied by Dr. John Mascola.

### Production of pseudoviruses in 293T cells

Wildtype or mutant pCAGGS SF162 gp160 plasmids were used to co-transfect 293T human embryonic kidney cells (ATCC, Manassas, VA, USA) with a Δenv backbone plasmid (pNL4.3Δenv or pSG3Δenv) at optimal backbone to envelope ratios, using Fugene HD (Roche, Manheim, Germany) according to the manufacturer's protocol. After 48–72 h, supernatants containing the secreted mutant or wildtype pseudoviruses were harvested, filtered, aliquoted and stored at −80°C. Before use in binding assays, pseudovirus supernatants were lysed by mixing with 0.1 volumes of 10% Triton X-100 in PBS.

### HIV Lysate Binding Assay

Binding Assays were carried out as previously described [27]. In brief, ELISA plates were coated overnight at 4°C with 1 µg/ml of sheep anti-HIV gp120 C5 (Aalto Bio Reagents Ltd., Dublin, Ireland), washed with PBS 0.05% Tween, and incubated for 1.5 h at 37°C with wildtype and mutant pseudovirus lysates. Plates were again washed and 10 µg/ml of conformational V2 mAbs (697, 830A, 1357D, 1361, 1393A, 2158, and 2297) or control antibodies (1418 and CD4-IgG2) were added for 1.5 h at 37°C. After washing, the bound mAbs were detected by 1.5 h, 37°C incubation with 1 µg/ml alkaline phosphatase (AP)-conjugated goat anti-human IgG(Fc) (Sigma-Aldrich, Inc., St. Louis, MO) followed by incubation with 1 mg/ml pNPP in Diethanolamine buffer. The plates were read after 20 min at 405 nm. All experiments were repeated a minimum of three times.

### Normalization of data

Binding (OD405 readings) of each conformational V2 mAb to each mutant pseudovirus was normalized to the CD4-IgG2 binding (OD405) of the same mutant pseudovirus. This controlled for possible differences in virion numbers among the pseudovirus lysates. Binding of wildtype pseudovirus lysate was included on each plate and set as 100%.

### Human monoclonal antibodies

The conformational V2 mAbs used in this study (697, 830A, 1357D, 1361, 1393A, 2158, and 2297) were produced in this laboratory and have been described previously [7], [20], [28]–[30]. The negative control antibody, mAb 1418 is specific for parvovirus B19 [31] and the positive control fusion protein used was CD4-IgG2 (Progenics Pharmaceuticals, Inc., Tarrytown, NY) [17].

### Modeling of SF162 structure

A full length V1V2 homology model for SF162 was created by using the template crystal structure of the 1FD6-V1V2-scaffold complexed with Fab PG9 (PDB Code: 3U4E). The SF162 sequence was threaded over the V1V2 template coordinates using Rosetta 3.4 [32]. Short gaps and deletions in the template structure were accommodated for by using Rosetta's full atom relaxation mode. The larger missing regions template (V2 positions 177–190) were modeled using Rosetta 3.4's *de novo* loop modeling protocols [33]. Briefly, the missing amino acids are inserted into the structure by performing fragment insertions using Monte Carlo sampling, followed by an energy minimization using explicit representations of all atoms. The structure with the lowest Rosetta Energy Unit (REU) was kept as the final SF162 structure model.

## Results

**Figure 1:**
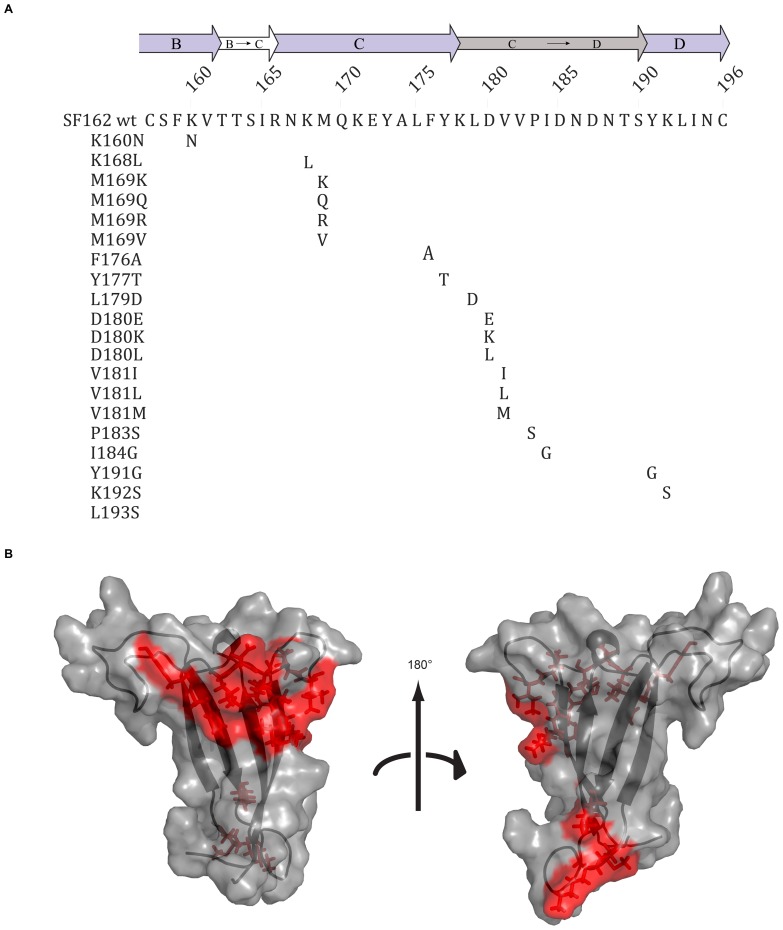
Mutations in the V2 region of SF162 used in epitope mapping studies. (A) The SF162 V2 wildtype sequence and a list of the mutations that were introduced at 13 different positions. The ß-strands (B, C, and D; purple) with the disordered region between the C and D β-strands (grey) are illustrated as arrows above the sequence. (B) The V1V2 domain of SF162, modeled after the structure published by McLellan et al.[4], indicating in red the amino acid where mutations were introduced.

Amino acids were exchanged by introducing point mutations at 13 different locations in the V2 region of the gp120 glycoprotein of SF162, starting at position 160 and continuing through position 193 (**Figure 1A and B**). Additionally, we introduced mutations at randomly selected positions in the C2 and C4 regions of gp120 as controls. The binding of seven anti-V2 mAbs (697, 830A, 1357D, 1361, 1393A, 2158, and 2297) to wildtype and mutant pseudovirus lysates was tested by ELISA. Monoclonal Ab 1418, specific for human parvovirus B19 [31], was included as a negative control in all experiments and did not bind to any of the pseudovirus lysates (data not shown). Binding of CD4-IgG2 [17] was used as a positive control and for normalization purposes, and was detected with all pseudovirus lysates tested (**Figure S1**). All values described are normalized to CD4-IgG2 and expressed as percentages of SF162 wildtype binding. Details are described in the Methods section.

As previous analysis had suggested that six of the seven conformational V2 mAbs share similar epitopes [20], changes were introduced at positions where the epitope of mAb 697 had originally been mapped [7]. We also made amino acid substitutions at positions 169 and 181, as these two locations were associated with vaccine-induced immune responses during the RV144 trial [3]. In addition, the lysine found at position 160 in SF162 was replaced with the more commonly represented residue at this position, asparagine, in order to reconstitute the critical glycan that has been shown to determine reactivity of the V1V2V3 quaternary mAbs [21], [34], [35]. Amino acid changes were introduced that occur at these positions in nature. The rationale behind all mutations can be found in **Table S1**.

### Mapping the binding epitope of mAb 697

**Figure 2:**
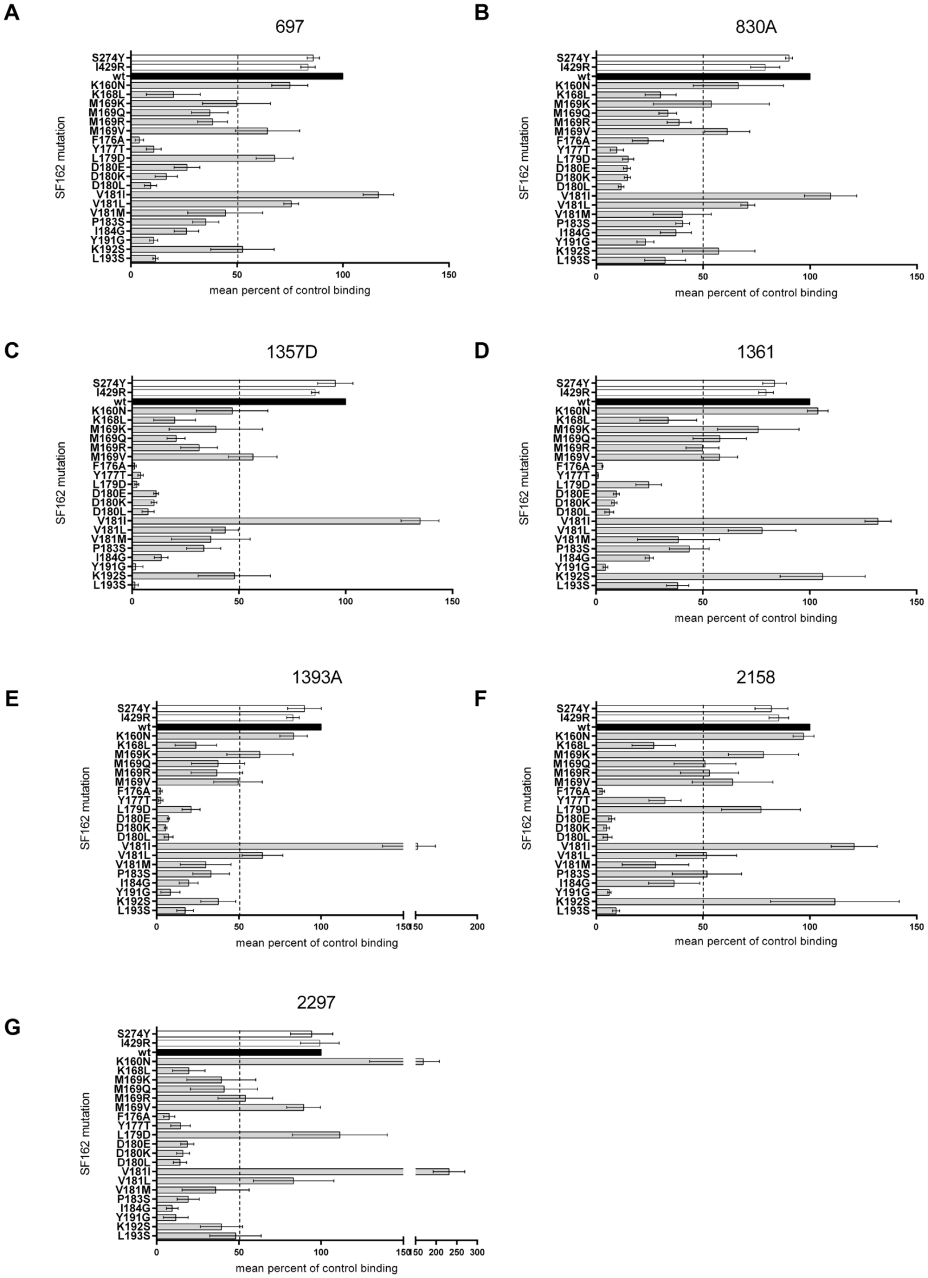
Binding of conformational V2 mAbs to wildtype and mutant SF162 pseudovirus lysates. Binding of (A) mAb 697, (B) mAb 830A, (C) mAb 1357D, (D) mAb 1361, (E) mAb 1393A, (F) mAb 2158, and (G) mAb 2297 to wildtype and 22 mutant SF162 pseudovirus lysates. The residue at each position in SF162 and the amino acid to which it was mutated is shown for each pseudovirus on the y-axis. Binding levels are shown on the x-axis; they are normalized to CD4-IgG2 binding (as described in Methods) and expressed as percentages of SF162 wildtype binding (black; 100%). The means of 3–5 experiments are shown with standard error of the mean.

Using HxB2 gp120 mutants, the epitope of mAb 697 was previously mapped to positions spanning residues between 176 and 193 in the V2 loop [7]. Our studies included mAb 697 in order to compare the epitope it recognized in HxB2 to that in SF162, to extend information about the epitope it recognizes, and to compare the epitopes of other conformational V2 mAbs to the epitope of mAb 697. An abrogation of binding to ≤15% compared to wildtype was observed for mutations F176A, Y177T, D180L, Y191G and L193S. Amino acid substitutions that reduced the binding of mAb 697 to ≤50% of wildtype included K168L, M169K, M169Q, M169R, D180E, D180K, V181M, P183S, and I184G. One mutation, V181I, increased binding slightly to 117%. All other changes in V2 reduced the binding by less than 50% (**Figure 2A** and **Table 1**). The mutations included as controls, in C2 and C4, had little effect on the binding of mAb 697: S274Y and I429R reduced the binding to 86% and 84% of wildtype SF162, respectively (**Figure 2A** and **Table 1**).

**Table 1:** Summary of binding of conformational V2 mAbs to wildtype and mutant SF162 pseudovirus lysates.

	697	830A	1357D	1361	1393A	2158	2297
**Wt**	100	100	100	100	100	100	100
**K160N**	75	66	47	104	83	97	169
**K168L**	20	30	20	34	24	27	20
**M169K**	50	54	39	76	63	78	39
**M169Q**	37	34	21	58	37	51	41
**M169R**	39	39	31	50	37	53	54
**M169V**	64	61	57	58	50	64	89
**F176A**	4	24	1	3	2	3	8
**Y177T**	11	10	4	1	2	32	14
**L179D**	68	15	2	25	21	77	111
**D180E**	26	14	11	10	7	7	19
**D180K**	17	15	10	9	5	5	16
**D180L**	8	11	8	6	7	6	14
**V181I**	117	110	135	132	152	121	231
**V181L**	76	71	44	78	64	52	83
**V181M**	45	40	37	39	30	28	36
**P183S**	35	40	34	44	33	52	19
**I184G**	26	37	14	25	19	37	9
**Y191G**	13	34	2	4	9	6	16
**K192S**	77	88	48	106	54	112	55
**L193S**	15	47	1	38	22	9	56
**C2 mutation (S274Y)**	86	90	95	84	90	82	94
**C4 mutation (I429R)**	84	79	86	80	83	86	99

* The values are normalized to CD4-IgG2 (as described in Methods) and expressed as percentages of SF162 wildtype binding (100%). The means of 3–5 experiments are shown.

### Mapping the binding epitope of mAb 830A

Like mAb 697, mAb 830A was developed from a subtype B infected individual using recombinant gp120_LAI_ and appeared, from previous data, to target an epitope similar to that of mAb 697 [20], [30]. Four amino acid substitutions abrogated binding of mAb 830A to ≤15% compared to wildtype (Y177T, L179D, D180E, D180K, and D180L). A reduction to ≤50% of wildtype was observed for mutations K168L, M169Q, M169R, F176A, V181M, P183S, I184G, Y191G and L193S whilst one mutation (V181I) led to an increase in binding. Mutations K160N, M169K, M169V, V181L, and K192S induced a change of <50% compared to wildtype binding (**Figure 2B** and **Table 1**). Binding of mAb 830A to pseudoviruses mutated in C2 (S274Y) and C4 (I429R) was 90% and 79%, respectively (**Figure 2B** and **Table 1**).

### Mapping the binding epitopes of mAbs 1357D, 1361, and 1393A

Monoclonal Abs 1357D, 1361, and 1393A were originally generated from the same patient by selection with oligomeric gp140_451_. Sequence analysis indicated that mAbs 1361 and 1393A are clonal [20]. As would be expected, these three mAbs displayed similar binding patterns with only subtle differences between them. Mutations that abrogated the binding of all three mAbs to ≤15% compared to wildtype included F176A, Y177T, D180E, D180K, D180L, and Y191G. Binding of mAbs 1357D, 1361, and 1393A was reduced to ≤50% by mutations K168L, M169R, V181M, P183S, and I184G. Mutations at only two positions induced slightly different binding results between these three mAbs: a change at position 179 from L to D led to an abrogation of binding of mAb 1357D to 2% whereas binding of mAbs 1361 and 1393A was reduced to 25% and 21%, respectively. Similarly, L193S abrogated binding of mAb 1357D to 1% but reduced binding of mAbs 1361 and 1393A to 38% and 22%, respectively (**Figures 2C, 2D, 2E** and **Table 1**). The binding of mAbs 1357D, 1361, and 1393A was increased to 135%, 132% and 152%,respectively, by replacing the V at position 181 with an I. Changes outside the V2 loop in C2 (S274Y) and C4 (I429R) resulted in 80–95% of wildtype binding (**Figures 2C, 2D, 2E** and **Table 1**).

### Mapping the binding epitope of mAb 2158

As opposed to the mAbs described above, mAb 2158, was derived from cells of a subtype B infected patient by selection with a V1V2-gp70 fusion protein carrying the V1V2 region from a clade B virus [36]. Despite this difference in selection, the binding pattern of this mAb was similar to those of the previously described mAbs, with very weak binding (relative to wildtype) for pseudoviruses carrying mutations at F176A, D180E, D180K, D180L, Y191G, or L193S [20], [30]. Similarly, substitutions K168L, Y177T, V181M, and I184G reduced the reactivity of mAb 2158 to ≤50% of wildtype, whilst two mutations (V181I and K192S) led to a slight increase in binding (121% and 112%, respectively). All other mutations induced a change of less than 50% compared to wildtype binding (**Figure 2F** and **Table 1**). Again, the control mutations S274Y and I429R induced little change in binding: 82% and 86% of wildtype binding, respectively (**Figure 2F** and **Table 1**).

### Mapping the binding epitopes of mAb 2297

Based on competition assays between mAbs 697 and 2297 for binding to V1V2-gp70, the epitopes recognized by these two mAbs appear to be different but overlapping [20]. Nonetheless, we found that mAb 2297 exhibited a similar binding pattern to mutant SF162 pseudovirus lysates compared to the other V2 mAbs tested. Binding was abrogated to ≤15% by 4 mutations – F176A, Y177T, D180L, and I184G, whilst a reduction in binding to ≤50% of wildtype was observed when mutations K168L, M169K, M169Q, D180E, D180K, V181M, P183S, and Y191G were introduced. Of the three mutations that increased binding of mAb 2297 (K160N, L179D, and V181I), V181I caused a particularly high increase, to 231% of wildtype binding, a level not detected with any other mAb. Binding of mAb 2297 was not affected by the control mutations at C2 (S274Y: 94%) and C4 (I429R: 99%) (**Figure 2G** and **Table 1**). Overall, mAb 2297 exhibited the lowest binding levels of all the mAbs to all of the tested pseudovirus lysates, including the wildtype SF162 (data not shown).

The data demonstrate that all seven mAbs target similar epitopes (**Figure 2** and **Table 1**), despite the differences in their VH gene usage and sequence differences [20]. Of particular interest is the finding that the V181I mutation *increases* binding for each of the seven mAbs. In contrast, V181L and V181M resulted in reduced binding by all mAbs. This position is part of the putative tri-peptide α4β7 integrin binding motif [11] which occurs at positions 179–181 in the midloop region of V2. Isoleucine is the most frequent residue at this position, and valine is the second most frequent residue. Mutations tested at positions 179 and 180 also resulted in reduced binding. The data suggest that the conformational V2 mAbs might target the α4β7 integrin binding motif, and that there is preferential binding of these mAbs to Envs that carry the isoleucine as part of this motif. In addition to residues 179–181, the binding of all seven conformational V2 mAbs appears to be particularly sensitive to changes in target amino acids at positions 176 and 177, N-terminal to the α4β7 integrin binding motif. Similarly, the binding of these mAbs is particularly sensitive to mutations at 191 and 193 (**Figure 2** and **Table 1**).

## Discussion

The V2 region of HIV-1 gp120 has been the subject of much study, having been shown to be a global regulator of neutralization sensitivity [30] and to differ in length and extent of glycosylation between transmitter/founder viruses and viruses of chronically infected patients [20], [37], [38]. However, V2 was not seriously considered to be a vaccine target until the results of the RV144 clinical vaccine trial showed that elevated levels of antibodies to the V1V2 region were associated with a reduced risk of infection and that two sites in V2 were under selective pressure in vaccinees [2], [3], [39].This has led to a renewed interest in antibodies that target V2 epitopes, including the only well-characterized human V2-specific mAbs which target conformation-dependent regions in V2 [7], [20], [28]–[30]. Since the epitopes of these latter seven mAbs had not been thoroughly mapped, we undertook this project, studying the binding of these mAbs to lysates of pseudoviruses carrying single amino acid substitutions in residues across the V2 loop. For these studies, we introduced amino acids that occur in nature at these positions.

**Figure 3:**
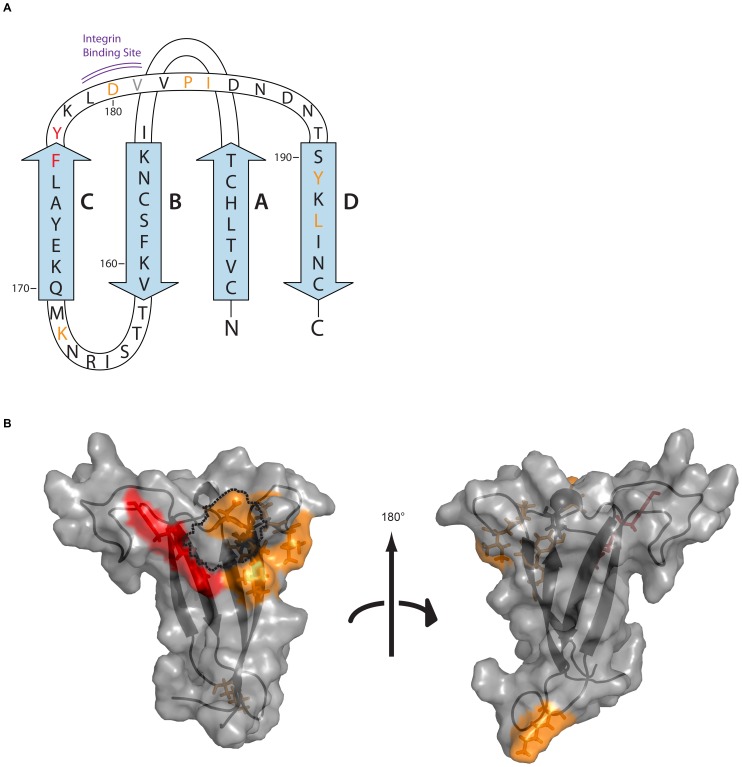
Location of conformational V2 epitopes and the effects of mutations. The V1V2 region of SF162 is illustrated as (A) a schematic diagram, and as (B) a 3D model where the effect of amino acid substitutions on binding is indicated and coded by color: Red: residues where binding of at least six conformational V2 mAbs was reduced to ≤15% of binding to wildtype; Orange: binding of at least six conformational V2 mAbs where binding was reduced to ≤50% of wildtype; Dark grey: effect dependent on amino acid substitution. Dashed lines outline the surface area of the LDV integrin binding site.

For all seven mAbs studied, specificity appeared to be particularly dependent on residues at positions 176–181 and at positions 191 and 193 (**Figure 2** and **Table 1**). Residues 176–181 are mostly located in the region disordered in the crystal structure of the scaffolded V1V2 in complex with mAb PG9, between the C and D β-strands of V2; positions 191 and 193 are found on the D β-strand (**Figure 3A**). Whilst these residues are discontinuous in a schematic two-dimensional diagram, in a three-dimensional representation modeled using the structure published by McLellan et al. [4], these amino acids cluster on the surface of V2, surrounding and overlapping the α4β7 integrin binding motif (**Figure 3B**).

**Figure 4:**
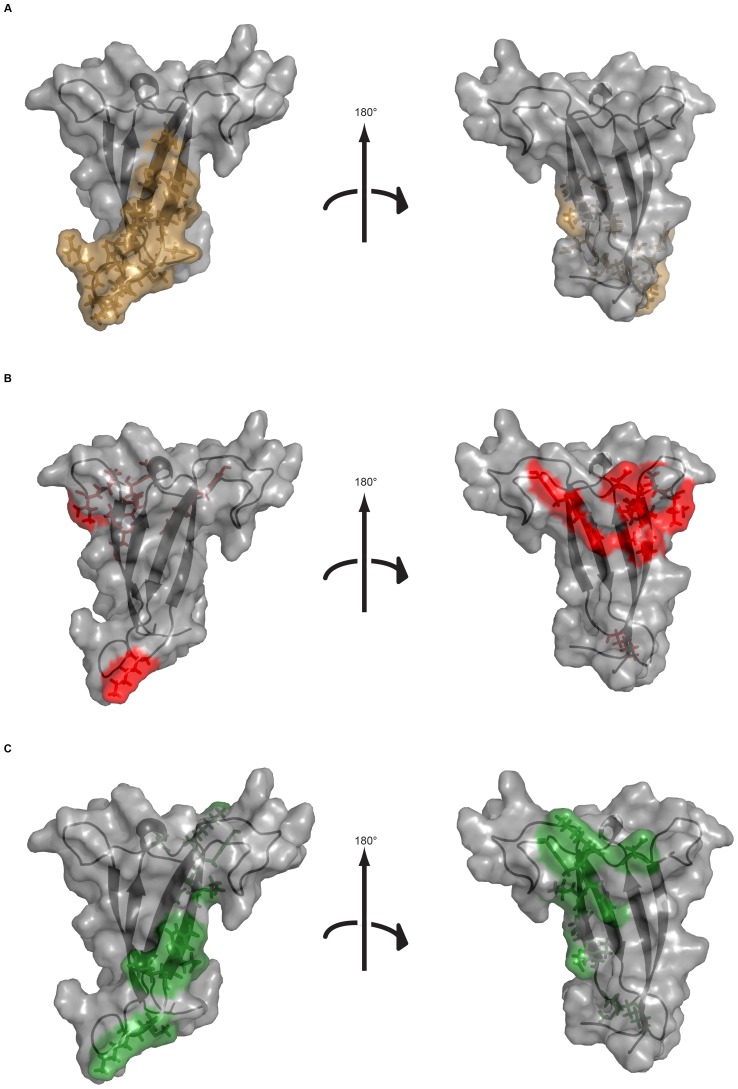
Comparison of the epitope surface areas of PG9 and the conformational V2 mAbs. Epitopes mapped onto the SF162 model of V1V2 for (A) PG9 (epitope shown in brown), (B) 697, 830A, 1361, 1393A, 1357D, 2158 and 2297 (epitope shown in red), (C) CH58/CH59 (epitope shown in green).

It is noteworthy that the binding of the conformational V2 mAbs is significantly affected by a mutation at position 168 which, in the 3D model, is located distant from the surface patch to which the other mutations map (**Figure 4B**), with the 168 residue appearing on the other side and at the bottom tip of the structure. This apparent discrepant positioning may be resolved by appreciating that the current model of V1V2 is a crystal structure in which V1V2 is held in a single conformation by mAb PG9. It is well-recognized, however, that the variable regions are highly disordered and flicker between several conformations [4], [22]. Alternative modeling of other possible conformations suggests that the C β-strand can form a helix bringing the Lysine at 168 in proximity to the epitope mapped for the conformational V2 mAbs (Spurrier et al., in preparation).

These mutagenesis-based epitope mapping can identify both residues which make direct contact with the antibody combining site and residues which, when mutated, alter the structure of the epitope but do not make contact with the antibody. Since the modeling studies included here indicate that many of the identified amino acids form a contiguous patch on the surface of V2, there is a high likelihood that most make direct contact with the antibody combining sites. However, further detailed computational analyses and crystallographic studies are on-going to determine which of the residues identified make antibody contact and which alter the epitope structure. Even though the seven conformational V2 mAbs were derived from five patients and selected with three different reagents, they all display a similar binding pattern (**Figure 2** and **Table 1**), indicating that the epitope recognized is a preferential immunogenic site on V2, and suggesting that this region could therefore serve as part of an effective vaccine. The epitope of these seven conformational V2 mAbs maps to a surface that clearly differs from that recognized by mAb PG9, specific for the quaternary neutralizing epitope (QNE) formed by V1, V2 and V3 [4], and from mAbs CH58 and CH59 derived from an RV144 vaccinee [22]. The PG9 epitope in the V1V2 crystal includes residues in the V1 β-strand B, the V2 β-strand C as well as glycans at N156 and N160 (in CAP45) [4]; this epitope maps onto the opposite side of the V1V2 complex from that bound by the conformational V2 mAbs (**Figures 4A and B**). The epitopes of the RV144-derived V2-specific mAbs CH58 and CH59 have binding footprints that include residues in a linear stretch in the C β-strand of V2 (amino acids 167–181 for CH58 and residues 168-173 for CH59) [22] which assumes different conformations than that recognized by PG9. Thus, given that QNE-specific mAbs preferentially bind to trimeric forms of Env [21], that RV144-derived mAbs can bind to peptides [22], that conformational V2 mAbs are highly cross-reactive with monomeric gp120 from diverse strains and fail to react with peptides [7], and that each of these V2-reactive mAbs generates a distinct epitope map (**Figures 4A, B and C**), the data show that there are (at least) three distinct types of V2-directed antibody responses.

## Supporting Information

Figure S1 **Binding of CD4-IgG2 to wildtype and mutant SF162 pseudovirus lysates.** The residue at each position in SF162 and the amino acid to which it was mutated is shown for each pseudovirus on the y-axis. Binding levels are shown on the x-axis and are expressed as percentages of SF162 wildtype binding (black; 100%). The means of 3-5 experiments are shown with the standard errors of the mean. White bars show mutations in C2 and C4 regions of gp120 and were used as controls.
(TIF)

Table S1 Mutations used for V2 mapping of monoclonal antibodies.
(DOCX)
