# Supplementary figures and images for: Epitope Mapping of Conformational V2-specific Anti-HIV Human Monoclonal Antibodies Reveals an Immunodominant Site in V2
Source: PLoS One. 2013 Jul 29;8(7):e70859. doi: 10.1371/journal.pone.0070859 (PMC3726596; doi:10.1371/journal.pone.0070859)

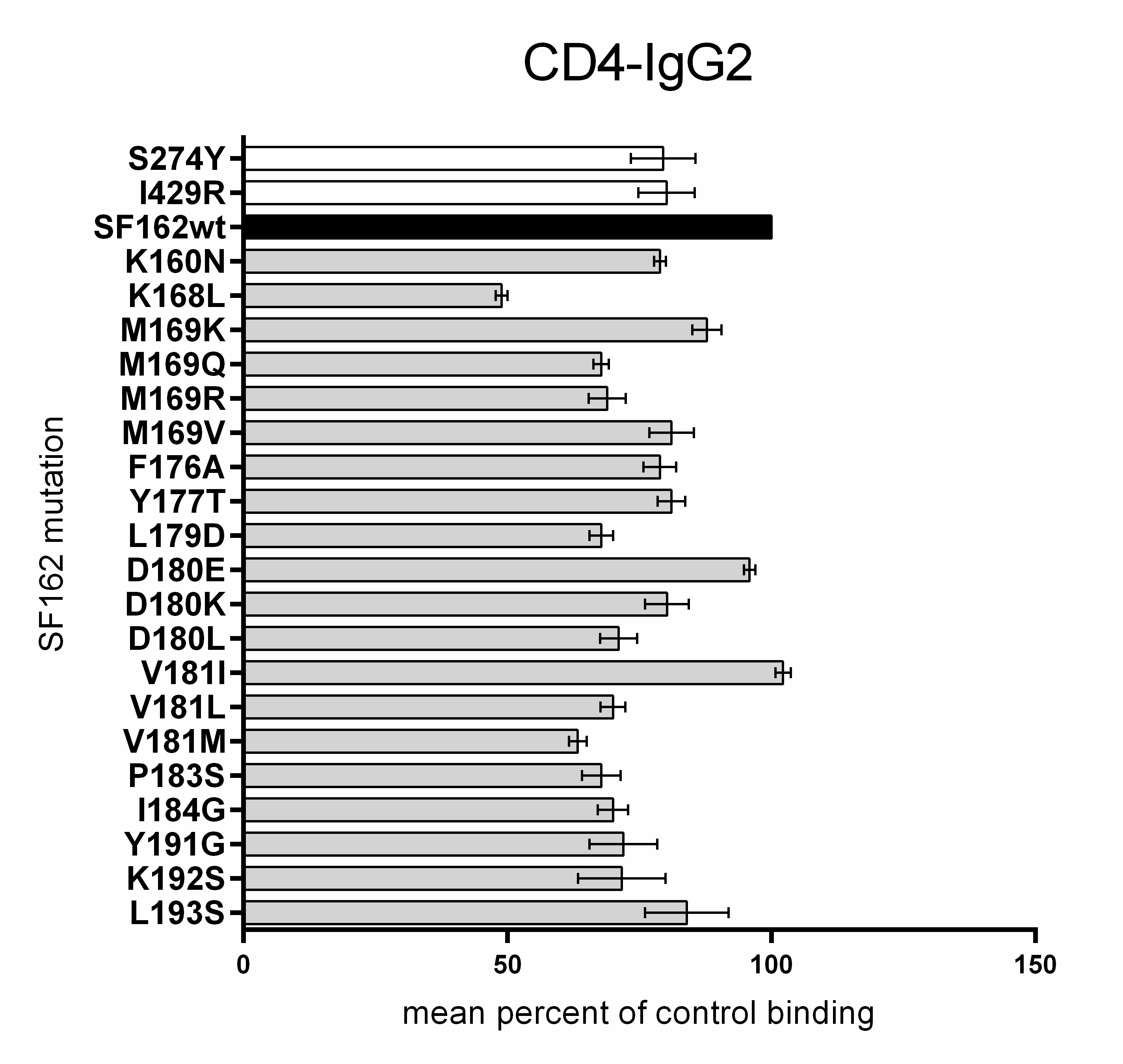

Supplement: Figure S1 — Binding of CD4-IgG2 to wildtype and mutant SF162 pseudovirus lysates. The residue at each position in SF162 and the amino acid to which it was mutated is shown for each pseudovirus on the y-axis. Binding levels are shown on the x-axis and are expressed as percentages of SF162 wildtype binding (black; 100%). The means of 3-5 experiments are shown with the standard errors of the mean. White bars show mutations in C2 and C4 regions of gp120 and were used as controls. (TIF) [file pone.0070859.s001.tif]
