# Supplementary material for: Epitope Mapping of Conformational V2-specific Anti-HIV Human Monoclonal Antibodies Reveals an Immunodominant Site in V2
Source: PLoS One. 2013 Jul 29;8(7):e70859. doi: 10.1371/journal.pone.0070859 (PMC3726596; doi:10.1371/journal.pone.0070859)
Supplement: Table S1 — Mutations used for V2 mapping of monoclonal antibodies. (DOCX) [file pone.0070859.s002.docx]

| Position | Residue | Mutation | Rationale |
| --- | --- | --- | --- |
| 160 | K | N | N is the most common residue at this position and the presence of an N-linked glycan at this position determines the specificity of quaternary mAbs ([21](#_ENREF_21), [34](#_ENREF_34), [35](#_ENREF_35)) |
| 168 | K | L | Gorny et al.([7](#_ENREF_7)), mutation reduces binding to gp120 HxB2 mutant by 52% |
| 169 | M | K | Present in Subtype AE A244 immunogen used in the RV144 clinical vaccine trial, and most frequent amino acid at this position |
|  |  | R | Rolland et al.([3](#_ENREF_3)), mutation associated with vaccine breakthrough viruses in sieve analysis of data from RV144 clinical trial |
|  |  | Q | Rolland et al.([3](#_ENREF_3)), mutation associated with vaccine breakthrough viruses in sieve analysis of data from RV144 clinical trial |
|  |  | V | Present in V1V2_Case A2_-gp70 fusion protein ([36](#_ENREF_36)), and 2^nd^ most frequent amino acid at this position |
| 176 | F | A | Gorny et al.([7](#_ENREF_7)), mutation at 176/177 FY>AT reduces binding to gp120 HxB2 mutant by 100% |
| 177 | Y | T | Gorny et al.([7](#_ENREF_7)), mutation at 176/177 FY>AT reduces binding to gp120 HxB2 mutant by 100% |
| 179 | L | D | Gorny et al.([7](#_ENREF_7)), mutation at 179/180 LD>DL reduces binding to gp120 HxB2 mutant by 100% |
| 180 | D | E, K, L | Gorny et al.([7](#_ENREF_7)), mutation at 179/180 LD>DL reduces binding to gp120 HxB2 mutant by 100% |
| 181 | V | I | Rolland et al.([3](#_ENREF_3)), residue found at this position that was reduced risk of infection in sieve analysis of data from RV144 clinical trial;most frequent amino acid at this position; and residue present in V1V2_Case A2_-gp70 ([36](#_ENREF_36)) and subtype AE A244 immunogen used in RV144 clinical vaccine trial |
|  |  | L | Rolland et al.([3](#_ENREF_3)), mutation found in sieve analysis of RV144 |
|  |  | M | Test replacement with other non-polar residue |
| 183 | P | S | Gorny et al.([7](#_ENREF_7)), mutation at 183/184 PI>SG reduces binding to gp120 HxB2 mutant by 90% |
| 184 | I | G | Gorny et al.([7](#_ENREF_7)), mutation at 183/184 PI>SG reduces binding to gp120 HxB2 mutant by 90% |
| 191 | Y | G | Gorny et al.([7](#_ENREF_7)), mutation at 192-194 YSL>GSS reduces binding to gp120 HxB2 mutant by 100% |
| 192 | K | S | Gorny et al.([7](#_ENREF_7)), mutation at 192-194 YSL>GSS reduces binding to gp120 HxB2 mutant by 100% |
| 193 | L | S | Gorny et al.([7](#_ENREF_7)), mutation at 192-194 YSL>GSS reduces binding to gp120 HxB2 mutant by 100% |
